# 2,2′-[(*E*,*E*)-*cis*-(Cyclo­hexane-1,4-di­yl)bis­(nitrilo­methanylyl­idene)]diphenol

**DOI:** 10.1107/S1600536812023367

**Published:** 2012-05-26

**Authors:** Shaaban K. Mohamed, Mehmet Akkurt, Muhammad N. Tahir, Antar A. Abdelhamid

**Affiliations:** a Chemistry and Environmental Division, Manchester Metropolitan University, Manchester M1 5GD, England; b Department of Physics, Faculty of Sciences, Erciyes University, 38039 Kayseri, Turkey; c University of Sargodha, Department of Physics, Sargodha, Pakistan

## Abstract

In the title compound, C_20_H_22_N_2_O_2_, the asymmetric unit contains two independent half-mol­ecules, which are both completed by crystallographic inversion symmetry. The cyclo­hexane rings of both mol­ecules adopt chair conformations; the N atoms are in equatorial orientations in one mol­ecule and in axial orientations in the other. Both mol­ecules feature two intra­molecular O—H⋯N hydrogen bonds, which generate *S*(6) rings.

## Related literature

For background to Schiff bases as ligands, see: Li & Zhang (2004 ▶).
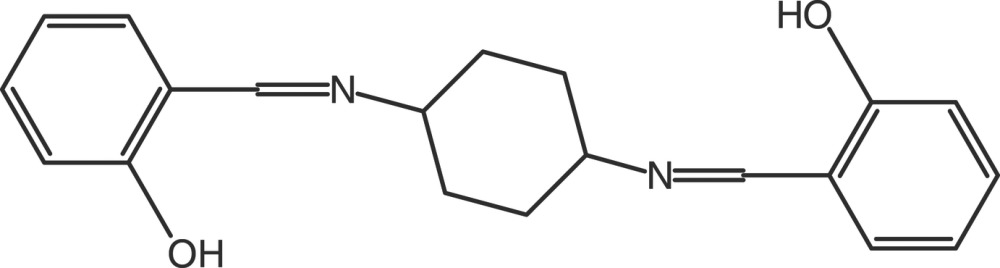


## Experimental

### 

#### Crystal data

C_20_H_22_N_2_O_2_
*M* *_r_* = 322.40
Monoclinic, 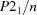

*a* = 16.2979 (11) Å
*b* = 6.1103 (4) Å
*c* = 18.2336 (12) Å
β = 104.975 (4)°
*V* = 1754.1 (2) Å^3^
*Z* = 4
Mo *K*α radiation
μ = 0.08 mm^−1^
*T* = 296 K
0.32 × 0.28 × 0.25 mm

#### Data collection

Bruker Kappa APEXII CCD diffractometer
Absorption correction: multi-scan (*SADABS*; Bruker, 2005 ▶) *T*_min_ = 0.975, *T*_max_ = 0.980
12904 measured reflections
3428 independent reflections
1641 reflections with *I* > 2σ(*I*)
*R*_int_ = 0.041

#### Refinement

*R*[*F* ^2^ > 2σ(*F* ^2^)] = 0.050
*wR*(*F* ^2^) = 0.140
*S* = 1.01
3428 reflections
219 parameters
H-atom parameters constrained
Δρ_max_ = 0.16 e Å^−3^
Δρ_min_ = −0.13 e Å^−3^

### 

Data collection: *APEX2* (Bruker, 2007 ▶); cell refinement: *SAINT* (Bruker, 2007 ▶); data reduction: *SAINT*; program(s) used to solve structure: *SHELXS97* (Sheldrick, 2008 ▶); program(s) used to refine structure: *SHELXL97* (Sheldrick, 2008 ▶); molecular graphics: *ORTEP-3 for Windows* (Farrugia, 1997 ▶) and *PLATON* (Spek, 2009 ▶); software used to prepare material for publication: *WinGX* (Farrugia, 1999 ▶) and *PLATON*.

## Supplementary Material

Crystal structure: contains datablock(s) global, I. DOI: 10.1107/S1600536812023367/hb6813sup1.cif

Structure factors: contains datablock(s) I. DOI: 10.1107/S1600536812023367/hb6813Isup2.hkl

Additional supplementary materials:  crystallographic information; 3D view; checkCIF report

## Figures and Tables

**Table 1:** Hydrogen-bond geometry (Å, °)

*D*—H⋯*A*	*D*—H	H⋯*A*	*D*⋯*A*	*D*—H⋯*A*
O1—H1⋯N1	0.82	1.85	2.579 (2)	148
O2—H2*A*⋯N2	0.82	1.86	2.593 (3)	148

**Fig. 1.**
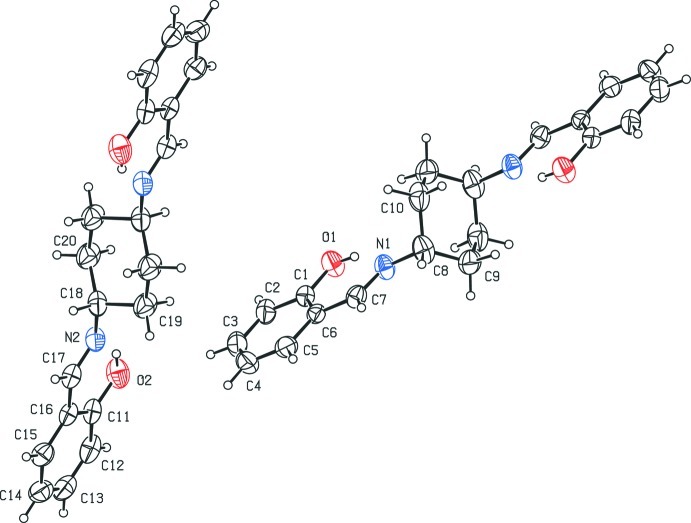
The molecular structure of the title compound, showing 30% probability ellipsoids.

**Fig. 2.**
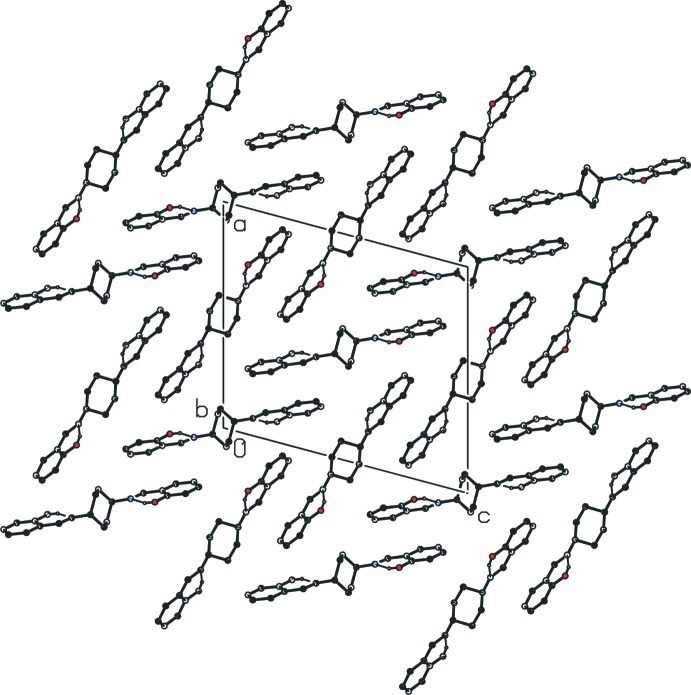
The crystal packing of the title compound, viewing along the b axis.

## supplementary crystallographic information

### Comment

Schiff base compounds have been reported as excelent substrates in the development of coordination chemistry (e.g. Li & Zhang, 2004), In this study we report the synthesis and crystal structure of the title compound (I).

As shown in Fig. 1, there are two independent half molecules A (with C1) and B (with C11) in the asymmetric unit of the title compound. They are centrosymmetric and the centres of symmetry are lied on the centroids of their cyclohexane rings. The cyclohexane rings of them adopt chair conformations Molecular conformation of the title compound is stabilized by intramolecular O—H···N hydrogen bonds, generating an S(6) ring motif (Table 1, Fig. 2).

### Experimental

The title compound arose as a bi-product from heating a reaction mixture of 114 mg (1 mmol) cyclohexane-1,4-diamine, 112 mg (1 mmol) cyclohexane-1,3-dione and 122 mg (1 mmol) salicylaldehyde in 50 ml e thanol under reflux for 6 h. The reaction mixture was concentrated under vacuum then left to cool at ambient temperature. The obtained solid was collected by Buckner funnel, washed with water then ethanol, dried in desiccator and crystallized from ethanol (m.p. 451 K). Yellow prisms were grown from ethanol solution by slow evaporation over two days.

### Refinement

All H atoms were positioned geometrically and allowed to ride on their parent atoms, with O—H = 0.82 Å and C—H = 0.93 Å (aromatic), 0.97 Å (methylene) and 0.98 Å (methine), with *U*_iso_(H) = 1.5*U*_eq_(O) for OH groups and *U*_iso_(H) = 1.2*U*_eq_(C) for others.

### Crystal data

**Table:**

C_20_H_22_N_2_O_2_	*F*(000) = 688
*M**_r_* = 322.40	*D*_x_ = 1.221 Mg m^−^^3^
Monoclinic, *P*2_1_/*n*	Mo *K*α radiation, λ = 0.71073 Å
Hall symbol: -P 2yn	Cell parameters from 355 reflections
*a* = 16.2979 (11) Å	θ = 3.5–18°
*b* = 6.1103 (4) Å	µ = 0.08 mm^−^^1^
*c* = 18.2336 (12) Å	*T* = 296 K
β = 104.975 (4)°	Prism, light yellow
*V* = 1754.1 (2) Å^3^	0.32 × 0.28 × 0.25 mm
*Z* = 4	

### Data collection

**Table:**

Bruker Kappa APEXII CCD diffractometer	3428 independent reflections
Radiation source: fine-focus sealed tube	1641 reflections with *I* > 2σ(*I*)
Graphite monochromator	*R*_int_ = 0.041
Detector resolution: 0.81 pixels mm^-1^	θ_max_ = 26.0°, θ_min_ = 2.3°
ω scans	*h* = −20→17
Absorption correction: multi-scan (*SADABS*; Bruker, 2005)	*k* = −7→7
*T*_min_ = 0.975, *T*_max_ = 0.980	*l* = −22→22
12904 measured reflections	

### Refinement

**Table:**

Refinement on *F*^2^	Primary atom site location: structure-invariant direct methods
Least-squares matrix: full	Secondary atom site location: difference Fourier map
*R*[*F*^2^ > 2σ(*F*^2^)] = 0.050	Hydrogen site location: inferred from neighbouring sites
*wR*(*F*^2^) = 0.140	H-atom parameters constrained
*S* = 1.01	*w* = 1/[σ^2^(*F*_o_^2^) + (0.0527*P*)^2^ + 0.1881*P*] where *P* = (*F*_o_^2^ + 2*F*_c_^2^)/3
3428 reflections	(Δ/σ)_max_ < 0.001
219 parameters	Δρ_max_ = 0.16 e Å^−^^3^
0 restraints	Δρ_min_ = −0.13 e Å^−^^3^

### Special details

**Table:**

Geometry. Bond distances, angles *etc*. have been calculated using the rounded fractional coordinates. All su's are estimated from the variances of the (full) variance-covariance matrix. The cell e.s.d.'s are taken into account in the estimation of distances, angles and torsion angles
Refinement. Refinement on *F*^2^ for ALL reflections except those flagged by the user for potential systematic errors. Weighted *R*-factors *wR* and all goodnesses of fit *S* are based on *F*^2^, conventional *R*-factors *R* are based on *F*, with *F* set to zero for negative *F*^2^. The observed criterion of *F*^2^ > σ(*F*^2^) is used only for calculating -*R*-factor-obs *etc*. and is not relevant to the choice of reflections for refinement. *R*-factors based on *F*^2^ are statistically about twice as large as those based on *F*, and *R*-factors based on ALL data will be even larger.

### Fractional atomic coordinates and isotropic or equivalent isotropic displacement parameters (Å^2^)

**Table:**

	*x*	*y*	*z*	*U*_iso_*/*U*_eq_	
O1	0.09064 (11)	−0.2099 (2)	0.21955 (9)	0.0798 (7)	
N1	0.07675 (13)	0.0983 (3)	0.12044 (10)	0.0689 (8)	
C1	0.13461 (14)	−0.0662 (4)	0.27110 (13)	0.0590 (9)	
C2	0.16439 (15)	−0.1325 (4)	0.34525 (13)	0.0737 (10)	
C3	0.20939 (17)	0.0070 (6)	0.39911 (15)	0.0845 (11)	
C4	0.22627 (17)	0.2171 (6)	0.37967 (16)	0.0878 (14)	
C5	0.19641 (15)	0.2847 (4)	0.30586 (15)	0.0732 (10)	
C6	0.15061 (13)	0.1469 (4)	0.25003 (12)	0.0540 (8)	
C7	0.11732 (14)	0.2225 (4)	0.17308 (13)	0.0629 (9)	
C8	0.0412 (2)	0.1898 (4)	0.04433 (14)	0.0798 (12)	
C9	0.07984 (17)	0.0747 (5)	−0.01193 (15)	0.0893 (13)	
C10	−0.05416 (19)	0.1620 (5)	0.02264 (14)	0.0895 (13)	
O2	0.24999 (12)	−0.0079 (3)	0.60105 (10)	0.0906 (8)	
N2	0.15493 (13)	0.3363 (3)	0.59279 (11)	0.0689 (8)	
C11	0.30811 (17)	0.0985 (4)	0.65534 (13)	0.0658 (10)	
C12	0.3858 (2)	−0.0030 (4)	0.68695 (16)	0.0789 (11)	
C13	0.44577 (18)	0.1016 (5)	0.74179 (17)	0.0835 (12)	
C14	0.43165 (18)	0.3068 (5)	0.76653 (15)	0.0827 (12)	
C15	0.35547 (17)	0.4075 (4)	0.73567 (14)	0.0726 (10)	
C16	0.29237 (15)	0.3078 (4)	0.67939 (13)	0.0594 (9)	
C17	0.21310 (16)	0.4199 (4)	0.64521 (13)	0.0639 (9)	
C18	0.07848 (17)	0.4641 (4)	0.56020 (12)	0.0707 (10)	
C19	0.08057 (16)	0.5442 (5)	0.48189 (14)	0.0877 (11)	
C20	0.00025 (17)	0.3308 (5)	0.55562 (15)	0.0893 (11)	
H1	0.07470	−0.14950	0.17810	0.0960*	
H2	0.15370	−0.27440	0.35880	0.0880*	
H3	0.22880	−0.03980	0.44920	0.1020*	
H4	0.25760	0.31200	0.41620	0.1050*	
H5	0.20730	0.42720	0.29300	0.0880*	
H7	0.12590	0.36770	0.16170	0.0760*	
H8	0.05480	0.34610	0.04490	0.0960*	
H9A	0.06250	0.14910	−0.06050	0.1070*	
H9B	0.14120	0.08380	0.00550	0.1070*	
H10A	−0.07710	0.22480	0.06200	0.1070*	
H10B	−0.07820	0.24110	−0.02410	0.1070*	
H2A	0.20730	0.06790	0.58720	0.1090*	
H12	0.39670	−0.14170	0.67070	0.0950*	
H13	0.49730	0.03250	0.76290	0.1000*	
H14	0.47330	0.37660	0.80380	0.0990*	
H15	0.34570	0.54600	0.75280	0.0870*	
H17	0.20470	0.55910	0.66260	0.0770*	
H18	0.07770	0.59130	0.59280	0.0850*	
H19A	0.08650	0.42010	0.45050	0.1050*	
H19B	0.12940	0.63890	0.48620	0.1050*	
H20A	−0.00150	0.28800	0.60640	0.1070*	
H20B	0.00280	0.19850	0.52690	0.1070*	

### Atomic displacement parameters (Å^2^)

**Table:**

	*U*^11^	*U*^22^	*U*^33^	*U*^12^	*U*^13^	*U*^23^
O1	0.1064 (14)	0.0639 (10)	0.0648 (11)	−0.0200 (10)	0.0146 (10)	0.0018 (8)
N1	0.0927 (15)	0.0592 (12)	0.0533 (12)	−0.0029 (11)	0.0160 (10)	0.0030 (10)
C1	0.0618 (15)	0.0655 (16)	0.0526 (14)	−0.0007 (13)	0.0202 (11)	−0.0056 (12)
C2	0.0842 (19)	0.0826 (18)	0.0583 (16)	0.0068 (15)	0.0257 (13)	0.0046 (14)
C3	0.084 (2)	0.114 (2)	0.0565 (17)	0.0167 (18)	0.0201 (14)	−0.0041 (17)
C4	0.0716 (19)	0.114 (3)	0.073 (2)	−0.0034 (17)	0.0099 (15)	−0.0310 (18)
C5	0.0669 (17)	0.0738 (17)	0.0834 (19)	−0.0097 (13)	0.0274 (14)	−0.0203 (15)
C6	0.0537 (14)	0.0567 (14)	0.0558 (14)	−0.0014 (11)	0.0217 (11)	−0.0057 (12)
C7	0.0736 (17)	0.0543 (14)	0.0672 (16)	−0.0008 (12)	0.0296 (13)	0.0013 (13)
C8	0.120 (3)	0.0562 (15)	0.0589 (16)	0.0001 (16)	0.0152 (16)	0.0100 (13)
C9	0.083 (2)	0.120 (3)	0.0661 (18)	−0.0034 (18)	0.0212 (14)	0.0239 (17)
C10	0.107 (3)	0.100 (2)	0.0625 (17)	0.0366 (19)	0.0238 (16)	−0.0014 (15)
O2	0.1192 (16)	0.0754 (12)	0.0774 (13)	0.0202 (11)	0.0259 (11)	−0.0102 (10)
N2	0.0781 (15)	0.0755 (14)	0.0541 (12)	0.0147 (12)	0.0189 (10)	0.0012 (11)
C11	0.085 (2)	0.0656 (17)	0.0548 (15)	0.0085 (15)	0.0327 (14)	0.0045 (13)
C12	0.101 (2)	0.0716 (18)	0.0787 (19)	0.0263 (18)	0.0497 (17)	0.0179 (15)
C13	0.073 (2)	0.104 (2)	0.084 (2)	0.0205 (18)	0.0391 (17)	0.0301 (18)
C14	0.067 (2)	0.097 (2)	0.088 (2)	−0.0028 (17)	0.0271 (15)	0.0135 (17)
C15	0.0768 (19)	0.0668 (16)	0.0809 (18)	−0.0025 (15)	0.0324 (15)	0.0048 (14)
C16	0.0693 (17)	0.0588 (15)	0.0594 (15)	0.0058 (13)	0.0335 (13)	0.0077 (12)
C17	0.0772 (18)	0.0605 (15)	0.0622 (16)	0.0092 (14)	0.0328 (13)	0.0061 (13)
C18	0.0829 (19)	0.0775 (17)	0.0530 (15)	0.0188 (16)	0.0202 (12)	0.0007 (13)
C19	0.0816 (19)	0.109 (2)	0.0774 (19)	0.0142 (17)	0.0296 (14)	0.0299 (16)
C20	0.089 (2)	0.104 (2)	0.0797 (19)	0.0131 (19)	0.0304 (15)	0.0309 (16)

### Geometric parameters (Å, º)

**Table:**

O1—C1	1.349 (3)	C9—H9A	0.9700
O1—H1	0.8200	C9—H9B	0.9700
O2—C11	1.347 (3)	C10—H10B	0.9700
O2—H2A	0.8200	C10—H10A	0.9700
N1—C7	1.267 (3)	C11—C16	1.397 (3)
N1—C8	1.469 (3)	C11—C12	1.394 (4)
N2—C18	1.460 (3)	C12—C13	1.363 (4)
N2—C17	1.267 (3)	C13—C14	1.372 (4)
C1—C6	1.401 (3)	C14—C15	1.370 (4)
C1—C2	1.374 (3)	C15—C16	1.392 (4)
C2—C3	1.363 (4)	C16—C17	1.453 (4)
C3—C4	1.378 (5)	C18—C20	1.497 (4)
C4—C5	1.371 (4)	C18—C19	1.518 (3)
C5—C6	1.382 (3)	C19—C20^ii^	1.523 (4)
C6—C7	1.443 (3)	C12—H12	0.9300
C8—C10	1.511 (5)	C13—H13	0.9300
C8—C9	1.509 (4)	C14—H14	0.9300
C9—C10^i^	1.504 (4)	C15—H15	0.9300
C2—H2	0.9300	C17—H17	0.9300
C3—H3	0.9300	C18—H18	0.9800
C4—H4	0.9300	C19—H19A	0.9700
C5—H5	0.9300	C19—H19B	0.9700
C7—H7	0.9300	C20—H20A	0.9700
C8—H8	0.9800	C20—H20B	0.9700
			
C1—O1—H1	109.00	H10A—C10—H10B	108.00
C11—O2—H2A	109.00	C8—C10—H10A	109.00
C7—N1—C8	119.3 (2)	O2—C11—C12	118.7 (2)
C17—N2—C18	119.0 (2)	C12—C11—C16	119.9 (2)
O1—C1—C2	118.9 (2)	O2—C11—C16	121.4 (2)
C2—C1—C6	120.0 (2)	C11—C12—C13	119.8 (2)
O1—C1—C6	121.1 (2)	C12—C13—C14	121.4 (3)
C1—C2—C3	120.8 (2)	C13—C14—C15	119.1 (3)
C2—C3—C4	120.2 (3)	C14—C15—C16	121.7 (2)
C3—C4—C5	119.3 (3)	C11—C16—C15	118.1 (2)
C4—C5—C6	121.8 (3)	C11—C16—C17	120.8 (2)
C1—C6—C5	117.9 (2)	C15—C16—C17	121.1 (2)
C1—C6—C7	120.9 (2)	N2—C17—C16	122.9 (2)
C5—C6—C7	121.1 (2)	N2—C18—C19	109.3 (2)
N1—C7—C6	122.4 (2)	N2—C18—C20	110.9 (2)
N1—C8—C10	109.4 (2)	C19—C18—C20	110.4 (2)
C9—C8—C10	110.6 (2)	C18—C19—C20^ii^	110.9 (2)
N1—C8—C9	109.4 (2)	C18—C20—C19^ii^	112.2 (2)
C8—C9—C10^i^	112.7 (2)	C11—C12—H12	120.00
C8—C10—C9^i^	112.0 (2)	C13—C12—H12	120.00
C1—C2—H2	120.00	C12—C13—H13	119.00
C3—C2—H2	120.00	C14—C13—H13	119.00
C4—C3—H3	120.00	C13—C14—H14	120.00
C2—C3—H3	120.00	C15—C14—H14	120.00
C3—C4—H4	120.00	C14—C15—H15	119.00
C5—C4—H4	120.00	C16—C15—H15	119.00
C6—C5—H5	119.00	N2—C17—H17	119.00
C4—C5—H5	119.00	C16—C17—H17	119.00
C6—C7—H7	119.00	N2—C18—H18	109.00
N1—C7—H7	119.00	C19—C18—H18	109.00
C9—C8—H8	109.00	C20—C18—H18	109.00
C10—C8—H8	109.00	C18—C19—H19A	109.00
N1—C8—H8	109.00	C18—C19—H19B	109.00
C8—C9—H9B	109.00	H19A—C19—H19B	108.00
C8—C9—H9A	109.00	C20^ii^—C19—H19A	109.00
C10^i^—C9—H9B	109.00	C20^ii^—C19—H19B	109.00
H9A—C9—H9B	108.00	C18—C20—H20A	109.00
C10^i^—C9—H9A	109.00	C18—C20—H20B	109.00
C8—C10—H10B	109.00	H20A—C20—H20B	108.00
C9^i^—C10—H10A	109.00	C19^ii^—C20—H20A	109.00
C9^i^—C10—H10B	109.00	C19^ii^—C20—H20B	109.00
			
C8—N1—C7—C6	177.0 (2)	N1—C8—C10—C9^i^	−67.2 (3)
C7—N1—C8—C9	119.4 (3)	C9—C8—C10—C9^i^	53.4 (3)
C7—N1—C8—C10	−119.3 (3)	C8—C9—C10^i^—C8^i^	54.6 (3)
C18—N2—C17—C16	−178.1 (2)	O2—C11—C12—C13	179.8 (3)
C17—N2—C18—C19	105.8 (3)	C16—C11—C12—C13	0.7 (4)
C17—N2—C18—C20	−132.2 (2)	O2—C11—C16—C15	−179.9 (2)
O1—C1—C2—C3	179.7 (2)	O2—C11—C16—C17	−1.4 (4)
C6—C1—C2—C3	0.2 (4)	C12—C11—C16—C15	−0.9 (4)
O1—C1—C6—C5	−179.7 (2)	C12—C11—C16—C17	177.7 (2)
C2—C1—C6—C7	−177.7 (2)	C11—C12—C13—C14	−0.4 (5)
O1—C1—C6—C7	2.8 (3)	C12—C13—C14—C15	0.4 (4)
C2—C1—C6—C5	−0.2 (3)	C13—C14—C15—C16	−0.6 (4)
C1—C2—C3—C4	−0.5 (4)	C14—C15—C16—C11	0.8 (4)
C2—C3—C4—C5	0.8 (4)	C14—C15—C16—C17	−177.7 (2)
C3—C4—C5—C6	−0.8 (4)	C11—C16—C17—N2	0.0 (4)
C4—C5—C6—C1	0.5 (4)	C15—C16—C17—N2	178.5 (2)
C4—C5—C6—C7	178.0 (2)	N2—C18—C19—C20^ii^	177.3 (2)
C1—C6—C7—N1	−4.2 (4)	C20—C18—C19—C20^ii^	55.0 (3)
C5—C6—C7—N1	178.4 (2)	N2—C18—C20—C19^ii^	−177.1 (2)
C10—C8—C9—C10^i^	−53.8 (3)	C19—C18—C20—C19^ii^	−55.7 (3)
N1—C8—C9—C10^i^	66.8 (3)	C18—C19—C20^ii^—C18^ii^	−56.0 (3)

Symmetry codes: (i) −*x*, −*y*, −*z*; (ii) −*x*, −*y*+1, −*z*+1.

### Hydrogen-bond geometry (Å, º)

**Table:**

*D*—H···*A*	*D*—H	H···*A*	*D*···*A*	*D*—H···*A*
O1—H1···N1	0.82	1.85	2.579 (2)	148
O2—H2*A*···N2	0.82	1.86	2.593 (3)	148
